# Complexes of Oligoribonucleotides with d-Mannitol Modulate the Innate Immune Response to Influenza A Virus H1N1 (A/FM/1/47) In Vivo

**DOI:** 10.3390/ph11030073

**Published:** 2018-07-22

**Authors:** Nataliia Melnichuk, Vladimir Kashuba, Svitlana Rybalko, Zenoviy Tkachuk

**Affiliations:** 1 Institute of Molecular Biology and Genetics, National Academy of Sciences of Ukraine, 03680 Kyiv, Ukraine; natalia.melnichuk8@gmail.com (N.M.); Vladimir.Kashuba@ki.se (V.K.); 2 Department of Microbiology, Tumor and Cell Biology (MTC), Karolinska Institute, S-17177 Stockholm, Sweden; 3 Gromashevsky L. V. Institute of Epidemiology and Infectious Diseases, NAMSU, 5 Amosov str., 03038 Kyiv, Ukraine; y_dasha@ukr.net

**Keywords:** oligoribonucleotides-d-mannitol complexes, influenza, pro-inflammatory immune responses

## Abstract

Rapid replication of the influenza A virus and lung tissue damage caused by exaggerated pro-inflammatory host immune responses lead to numerous deaths. Therefore, novel therapeutic agents that have anti-influenza activities and attenuate excessive pro-inflammatory responses that are induced by an influenza virus infection are needed. Oligoribonucleotides-d-mannitol (ORNs-d-M) complexes possess both antiviral and anti-inflammatory activities. The current research was aimed at studying the ORNs-d-M effects on expression of innate immune genes in mice lungs during an influenza virus infection. Expression of genes was determined by RT-qPCR and Western blot assays. In the present studies, we found that the ORNs-d-M reduced the influenza-induced up-expression of Toll-like receptors (TLRs) (*tlr3*, *tlr7*, *tlr8*), nuclear factor NF-kB (*nfkbia*, *nfnb1*), cytokines (*ifnε*, *ifnk*, *ifna2*, *ifnb1*, *ifnγ*, *il6*, *il1b*, *il12a*, *tnf*), chemokines (*ccl3*, *ccl4*, *сcl5*, *cxcl9*, *cxcl10*, *cxcl11*), interferon-stimulated genes (ISGs) (*oas1a*, *oas2*, *oas3*, *mx1*), and pro-oxidation (*nos2*, *xdh*) genes. The ORNs-d-M inhibited the mRNA overexpression of *tlr3*, *tlr7*, and *tlr8* induced by the influenza virus, which suggests that they impair the upregulation of NF-kB, cytokines, chemokines, ISGs, and pro-oxidation genes induced by the influenza virus by inhibiting activation of the TLR-3, TLR-7, and TLR-8 signaling pathways. By impairing activation of the TLR-3, TLR-7, and TLR-8 signaling pathways, the ORNs-d-M can modulate the innate immune response to an influenza virus infection.

## 1. Introduction

The influenza A virus causes pandemics, which makes it responsible for high mortality rates and great economic losses every year [1]. Currently, annual trivalent or quadrivalent vaccines are the main anti-influenza therapeutics. However, rapid antigenic drift and the shift in influenza viruses make it difficult to select appropriate vaccine strains [2,3,4,5]. Furthermore, the anti-influenza licensed drugs are currently limited to oseltamivir, zanamivir [6], amantadine, and rimantadine [7,8]. However, the emergence of drug-resistant influenza variants [8,9,10] and co-infection with influenza and other respiratory viruses decrease with regard to the efficiency of these drugs [11,12]. Therefore, new anti-influenza therapeutics with novel mechanisms of action are urgently required to combat the persistent threat of influenza viruses.

Infection by the influenza A virus is frequently characterized by considerable inflammation [13]. Lung tissue damage is a consequence of diseases associated with influenza A virus infections since inflammation results from the release of pro-inflammatory chemokines and the recruitment of neutrophils, lymphocytes, and particularly mononuclear phagocytes into the alveolar space to limit the viral spread [14]. Therefore, using a therapeutic drug to impair the innate immune response in combination with antiviral action has the potential to diminish symptoms and tissue damage caused by the influenza A virus infection [15]. Currently, it is important to search for anti-influenza drugs with a wide spectrum of antiviral action.

Aptamers are synthetic single-stranded DNA (ssDNA) or RNA sequences that can bind with high affinity and specificity to a wide range of target molecules such as proteins, cell surface receptors, and even whole cells [16,17] as well as other organic or inorganic molecules such as adenosine (also AMP and ATP), dyes, amino acids, drugs, or simple small cations [18]. Theoretically, aptamers can be used therapeutically in any disease [18,19,20]. 2′-5′-Oligoadenylates have antiviral actives and can bind to some proteins (interferon α, S100 calcium-binding protein A1, protein kinases), which changes the conformation of these proteins [21,22,23].

Complexes of natural oligoribonucleotides with d-mannitol (ORNs-d-M) based on total yeast RNA modified with d-mannitol (d-M) have a wide range of biological activities playing a key role in the antiviral activity and can be used as an anti-influenza drug [24,25]. These complexes were registered under the commercial name Nuclex in Ukraine. Previously, one study showed that intraperitoneal and intravenous injections of the ORNs-d-M into mice in doses from 15 mg/kg to 150 mg/kg have high antiviral activity against the influenza A virus H1N1 (A/FM/1/47) (FM147) infection [26]. Mechanisms of anti-influenza activity of the ORNs-d-M inhibited the neuraminidase (NA) activity [26] and hemagglutinin (HA)-glycan interaction of the influenza virus [27]. In our previous studies, we suggested that, besides inhibiting the activity of surface viral proteins (NA, HA), the ORNs-d-M can have another mechanism responsible for anti-influenza activity of these complexes [27]. The ORNs-d-M are known to possess an anti-inflammatory action [28,29,30,31]. Therefore, in this paper, we investigated the influence of the ORNs-d-M on up-expression of the innate immune genes induced by the influenza virus infection with the aim of studying other mechanisms of this drug as an anti-influenza therapeutic agent with an anti-inflammatory effect. In current research, we found that, during the influenza virus FM147 infection, the ORNs-d-M efficiently impair activation of Toll-like receptors (TLR) 3, 7, 8 signaling pathways, interfere up-expression of TLRs (*tlr3*, *tlr7*, *tlr8*), nuclear factor NF-kB (*nfkbia*, *nfnb1*), cytokines (*ifnε*, *ifnk*, *ifna2*, *ifnb1*, *ifnγ*, *il6*, *il1b*, *il12a*, *tnf*), chemokines (*ccl3*, *ccl4*, *сcl5*, *cxcl9*, *cxcl10*, *cxcl11*), interferon-stimulated genes (ISGs) (*oas1a*, *oas2*, *oas3*, *mx1*), and pro-oxidation (*nos2*, *xdh*) genes that induced the infection.

## 2. Results

### 2.1. The ORNs-d-М Inhibit the Up-Expression of nos2, arg2, xdh Genes Induced by the Influenza Virus and Decrease the Level of Lipid Peroxidation Products in Lungs of Influenza-Infected Mice

**Figure 1:**
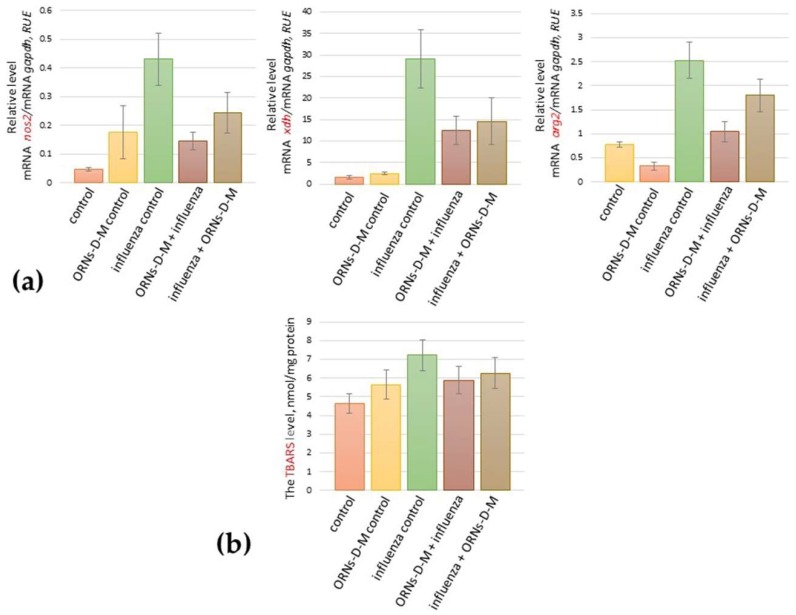
(**a**) Impair the up-regulation of *nos2*, *arg2*, and *xdh* induced by the influenza virus A/Fort Monmouth/1/1947-mouse adapted (H1N1) and (**b**) decrease the LPO products at the influenza virus infection owing to the ORNs-d-М in vivo. Before and after infection with the influenza virus FM147 (4.0 lg LD_50_), the BALB/c mice were treated with the ORNs-d-М. Total RNAs from the mice lungs were isolated and RT-qPCR was performed. The investigated mRNA levels were normalized to *gapdh* as a control. The TBARS level was tested as described by Asakawa and Matsushita. RUE: relative units of expression. ORNs-d-М control—ORNs-d-М injection into healthy mice, influenza control—infection of mice with the influenza virus, ORNs-d-М + influenza—ORNs-d-М injection 24 h before influenza virus infection as a form of prevention with ORNs-d-М, and influenza + ORNs-d-М—ORNs-d-М injection 24 h after influenza virus infection as treatment with ORNs-D-М. Data are shown as the mean ± SD for three independent experiments.

To determine the influence of the ORNs-d-М on up-expression of some pro-oxidation genes induced by the influenza virus, the mRNA levels of *nos2*, *arg2*, and *xdh* in mice lungs were determined by RT-qPCR after prevention and treatment with the ORNs-d-М of the influenza virus infection. As shown in Figure 1a, the overexpression of these investigated genes was detected in mice lungs 48 h after infection with the influenza virus when compared to the control. Conversely, the ORNs-d-М injection into healthy mice as a positive control of the ORNs-d-М, the mRNA expression levels of *nos2*, *arg2*, and *xdh* remained unchanged when compared to the healthy ones. The ORNs-d-М injection for prevention and treatment of the influenza infection reduced the mRNA level of *nos2*, *arg2*, and *xdh* expression in comparison with the virus-infected mice.

After 48 h of infection with the influenza virus and both prevention and treatment with the ORNs-d-М of the influenza infection, the level of lipid peroxidation (LPO) products in mice lungs was measured by thiobarbituric acid reactive species (TBARS). The level of TBARS in influenza-infected mice lungs was found to be 43% higher than the control while both prevention and treatment with ORNs-d-М of the influenza virus infection decreased the TBARS level by 18% and 15%, respectively, when compared to the infected mice (Figure 1b). An unchanged TBARS level was observed in mice lungs after the ORNs-d-М injection without the influenza virus infection and was compared to the control.

### 2.2. The ORNs-d-М Inhibit the Overexpression of Cytokines, Chemokines, and ISGs Induced by the Influenza Virus In Vivo

**Figure 2:**
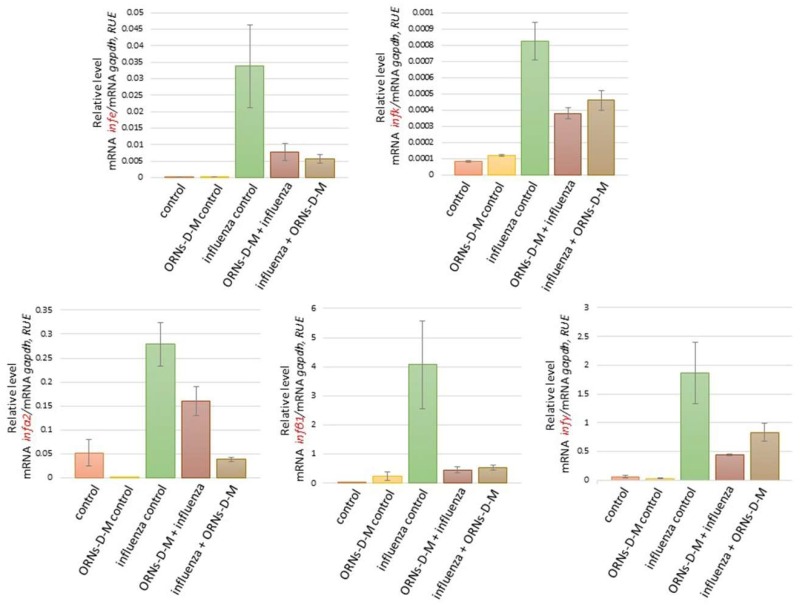
Impaired up-regulation of the cytokines *ifnε*, *ifnk*, *ifna2*, *ifnb1*, and *ifnγ* induced by the influenza virus A/Fort Monmouth/1/1947-mouse adapted (H1N1) owing to the ORNs-d-М in vivo. Before and after infection with the influenza virus FM147 (4.0 lg LD_50_), the BALB/c mice were treated with the ORNs-d-М. Total RNAs from the mice lungs were isolated and RT-qPCR was performed. The investigated mRNA levels were normalized to *gapdh* as a control. RUE: relative units of expression. ORNs-d-М control—ORNs-d-М injection into healthy mice, influenza control—infection of mice with influenza virus, ORNs-d-М + influenza—ORNs-d-М injection 24 h before influenza virus infection as prevention with ORNs-d-М, and influenza + ORNs-d-М—ORNs-d-М injection 24 h after influenza virus infection as treatment with ORNs-d-М. Data are shown as the mean ± SD for three independent experiments.

**Figure 3:**
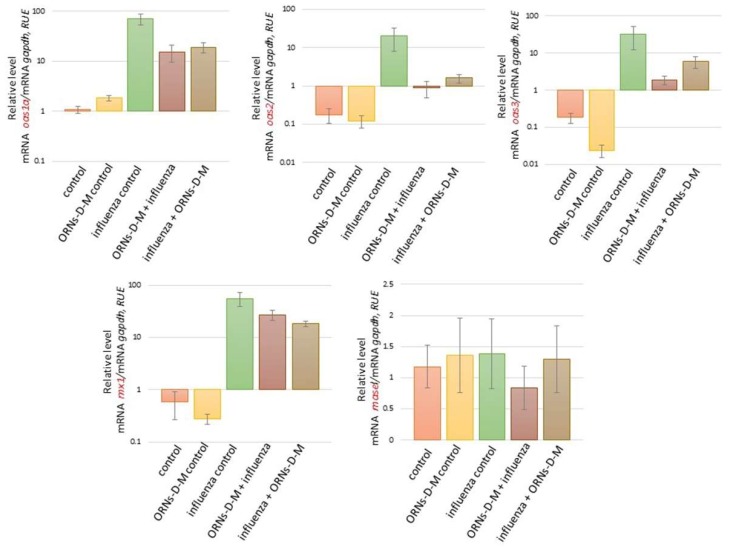
Impaired up-regulation of ISGs *oas1a*, *oas2*, *oas3*, *mx1*, and *rnasel* induced by the influenza virus A/Fort Monmouth/1/1947-mouse adapted (H1N1) owing to the ORNs-d-М in vivo. Before and after infection with the influenza virus FM147 (4.0 lg LD_50_), the BALB/c mice were treated with the ORNs-d-М. Total RNAs from the mice lungs were isolated and RT-qPCR was performed. The investigated mRNA levels were normalized to *gapdh* as a control. RUE: relative units of expression. ORNs-d-М control—ORNs-d-М injection into healthy mice, influenza control—infection of mice with an influenza virus, ORNs-d-М + influenza—ORNs-d-М injection 24 h before influenza virus infection as prevention with ORNs-d-М, and influenza + ORNs-d-М—ORNs-d-М injection 24 h after influenza virus infection as treatment with ORNs-d-М. Data are shown as the mean ± SD for three independent experiments.

**Figure 4:**
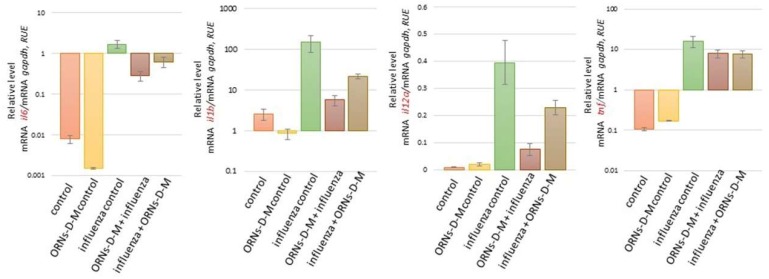
Impair the pro-inflammatory cytokines *il6*, *il1b*, *il12a*, and *tnf* overexpression induced by an influenza virus A/Fort Monmouth/1/1947-mouse adapted (H1N1) owing to the ORNs-d-М in vivo. Before and after infection with the influenza virus FM147 (4.0 lg LD_50_), the BALB/c mice were treated with ORNs-d-М. Total RNAs from the mice lungs were isolated and RT-qPCR was performed. Samples were normalized to *gapdh* as a control. RUE: relative units of expression. ORNs-d-М control—ORNs-d-М injection into healthy mice, influenza control—infection of mice with influenza virus, ORNs-d-М + influenza—ORNs-d-М injection 24 h before infection with the influenza virus and prevention with ORNs-d-М, and influenza + ORNs-d-М—ORNs-d-М injection 24 h after infection with influenza virus and treatment with ORNs-d-М. Data are shown as the mean ± SD for three independent experiments.

**Figure 5:**
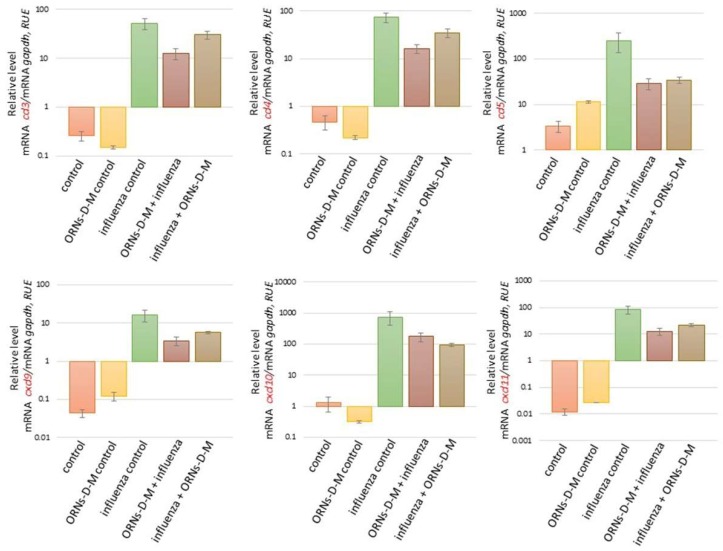
Impair the pro-inflammatory chemokines overexpression induced by the influenza virus A/Fort Monmouth/1/1947-mouse adapted (H1N1) owing to the ORNs-d-М in vivo. Before and after infection with the influenza virus FM147 (4.0 lg LD_50_), the BALB/c mice were treated with ORNs-d-М. Total RNA from the mice lung was isolated and RT-qPCR was performed. Samples were normalized to *gapdh* as a control. RUE: relative units of expression. ORNs-d-М control—ORNs-d-М injection into healthy mice, influenza control—infection of mice with influenza virus, and ORNs-d-М + influenza—ORNs-d-М injection 24 h before infection with the influenza virus and prevention with ORNs-d-М, and influenza + ORNs-d-М—ORNs-d-М injection 24 h after infection with the influenza virus and treatment with ORNs-d-М. Data are shown as the mean ± SD for three independent experiments.

We also investigated the influence of the ORNs-d-М on expression of cytokines, chemokines, and ISGs at the influenza virus infection. As shown in Figure 2 and Figure 3, the increased mRNA expression level of cytokines *ifnε*, *ifnk*, *ifna2*, *ifnb1*, and *ifnγ* and ISGs *oas1a*, *oas2*, *oas3*, and *mx1* was determined 48 h after influenza virus infection in comparison with the control. It was also detected to increase the mRNA level of the pro-inflammatory cytokines *il6*, *il1b*, *il12a*, and *tnf* and chemokines *ccl3*, *ccl4*, *сcl5*, *cxcl9*, *cxcl10*, and *cxcl11* induced by the influenza virus infection (see Figure 4 and Figure 5). However, unchanged mRNA expression of these investigated genes was observed in lungs of ORNs-d-М-treated mice without the influenza virus infection in comparison with the control (Figure 2, Figure 3, Figure 4 and Figure 5).

In addition, both prevention and treatment with the ORNs-d-М during the influenza virus infection led to a reduced mRNA level of *ifnε*, *ifnk*, *ifna2*, *ifnb1*, *ifnγ*, *oas1a*, *oas2*, *oas3*, *mx1* (Figure 4), *il6*, *il1b*, *il12a*, *tnf*, *ccl3*, *ccl4*, *сcl5*, *cxcl9*, *cxcl10*, and *cxcl11* (Figure 5) compared with the influenza control. Conversely, the *rnasel* mRNA expression remained unchanged in lungs of the infected mice, the treated mice with the ORNs-d-М without the influenza virus infection, and the treated mice with the ORNs-d-М for prevention and treatment of the influenza virus infection in comparison with the control (see Figure 3).

### 2.3. ORNs-d-М Inhibit the Overexpression of nfkb1, nfkbiα, tlr3, tlr7, and tlr8 Induced by the Influenza Virus

**Figure 6:**
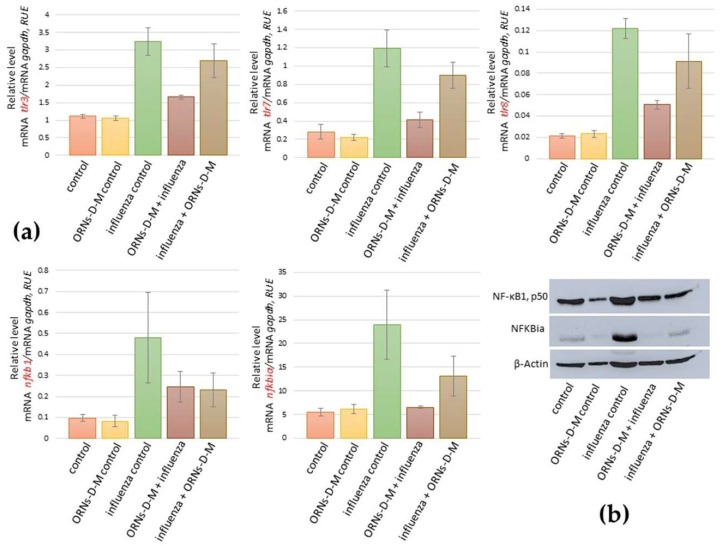
Impaired overexpression of the *tlr3*, *tlr7*, *tlr8*, *nfkb1*, and *nfkbiα* induced by the influenza virus A/Fort Monmouth/1/1947-mouse adapted (H1N1) owing to the ORNs-d-М *in vivo*. Before and after infection with the influenza virus FM147 (4.0 lg LD_50_), the BALB/c mice were treated with ORNs-d-М. (**a**) Total RNAs from the mice lungs were isolated and RT-qPCR was performed. The mRNA levels of *tlr3*, *tlr7*, *tlr8*, *nfkb1*, and *nfkbiα* were normalized to *gapdh* as a control. RUE: relative units of expression. ORNs-d-М control—ORNs-d-М injection into healthy mice, influenza control—infection of mice with influenza virus, ORNs-d-М + influenza—ORNs-d-М injection 24 h before infection with the influenza virus and prevention with ORNs-d-М, and influenza + ORNs-d-М—ORNs-d-М injection 24 h after infection with the influenza virus and treatment with ORNs-d-М. Data are shown as the mean ± SD for three independent experiments. (**b**) The investigated mice lungs were subjected to Western blot analysis. The protein levels of NF-kB1 and NFKBia were quantified by densitometric analysis and normalized to β-actin.

Investigating mRNA of the *tlr3*, *tlr7*, *tlr8*, *nfkbia*, and *nfnb1*, we found the same tendency as during studding of the mRNA level of *xdh*, *nos2*, *arg2*, *oas1a*, *oas2*, *oas3*, *mx1*, *ifnε*, *ifnk*, *ifna2*, *ifnb1*, *ifnγ*, *ccl3*, *ccl4*, *сcl5*, *cxcl9*, *cxcl10*, *cxcl11*, *il6*, *il1b*, *il12a*, and *tnf* (Figure 1a and Figure 2, Figure 3, Figure 4 and Figure 5). For example, it was shown that the mRNA of *nfkb1*, *nfkbiα*, *tlr3*, *tlr7*, and *tlr8* increased in mice lungs 48 h after infection with the influenza virus and compared to the control (see Figure 6a). However, the mRNA of *nfkb1*, *nfkbiα*, *tlr3*, *tlr7*, and *tlr8* after both prevention and treatment with ORNs-d-M of the influenza virus infection decreased vs. the influenza-infected mice. Additionally, unchanged mRNA expression of these investigated genes was observed in lungs of the ORNs-d-М-treated mice without the influenza virus infection and compared to the control.

By using a Western blot assay, protein levels of the *nfkb1*, *nfkbiα* were studied in mice lungs that had been infected with the influenza virus and had been treated with the ORNs-d-М for prevention and treatment of the influenza virus infection (see Figure 6b). The protein level of *nfkb1*, *nfkbiα* increased in the influenza-infected mice lungs when compared to the control while both ORNs-d-М prevention and treatment of the influenza infection reduced the protein level of *nfkb1* and *nfkbiα* compared to the influenza-infected mice. Unchanged protein expression of these genes was observed in lungs of the uninfected mice that had been treated with the ORNs-d-М in comparison with the control.

Additionally, the infectious titer of the influenza virus after prevention and treatment with the ORNs-d-М was investigated using the TCID_50_ assay. As shown in Table 1, 48 h after infection with the influenza virus, both prevention and treatment with the ORNs-d-М decreased the infectious titer of the influenza virus in comparison with the influenza control. Similar tendency was observed at studding of weight loss.

## 3. Discussion

The influenza virus induces lung tissue damage by causing overproduction of free radicals including reactive nitrogen intermediates (RNIs) (NO, NO_2_, HNO_2_) and reactive oxygen species (ROSs) (O_2_^−^, OH, H_2_O_2_^−^) [32]. During acute influenza virus infection, an increased level of free radicals can directly contribute to cell death of infected lung tissue and exacerbate pathology caused by the influenza virus replication [33]. The important pro-oxidation genes, which are responsible for generating free radicals, are *xdh* and *nos2* [34]. The pathogenic role of RNIs and ROSs during influenza virus infection realizes by increasing the enzyme activity of NOS, XO, and mRNA expression of *nos2* and *xdh* in influenza-infected lungs [34,35,36]. Arginase activation in the airway epithelial cells causes a reduction in *nos2* expression, which reduces NO generation [37]. In the presented study, we found the overexpression of *xdh*, *nos2*, and *arg2* genes induced by the influenza virus FM147 infection (Figure 1a).

In our previous studies, we found that the ORNs-d-М have antiviral activity against RNA and DNA viruses with a wide spectrum of antiviral action [24]. The total yeast RNA possesses an anti-inflammatory action and stabilize nitric oxide synthase (NOS) activity in vitro and in vivo [28,29,30,31]. In this study, we found that the ORNs-d-М injection for prevention and treatment reduced the *nos2*, *arg2*, and *xdh* up-expression induced by the influenza virus infection (Figure 1a).

Oxidative stress induced by overproduction of the free radical increases the LPO level during influenza virus H1N1 infection [38]. Next, we estimated an ability of the ORNs-d-М to affect the level of LPO products in mice lungs during the influenza virus infection and detected that the ORNs-d-М injection for prevention and treatment can decrease the level of LPO in influenza-infected mice (Figure 1b), which indicates that these ORNs-d-М likely decrease the protein level of *nos2*, *arg2*, and *xdh* during the influenza virus infection. These results suggest that the ORNs-d-М can impair the up-regulation of *nos2*, *xdh*, and *arg2* genes and increase LPO products induced by the influenza virus, which suppresses NOS activity and stabilizes the membrane [28,39].

The cytokines and chemokines are one of the main defenders against the virus infection, which involve inflammatory cells to the infection site [34]. Interferons (IFNs) are a multigene family of inducible cytokines [40,41,42], which possess an antiviral activity [43,44]. IFNs of type I that included the IFN-ε, IFN-k IFN-α, and IFN-β are known as viral IFNs and IFN of type II (IFN-γ) is known as immune IFN [43,45]. IFN-β is expressed within 3–6 h of influenza infection in airway epithelial cells [46,47]. IFN-β initiates the antiviral genes transcription including RNA-dependent protein kinase, 2′,5′-oligoadenylate synthetase (OAS), and RNase L, Mx protein, and GTPases in neighboring cells [48,49,50]. Influenza infection also induces the production of such cytokines as TNFa, IL-1, IL-6, and the mononuclear cell attractant chemokines: CCL-3, CCL-4, CCL-5, CXCL9, CXCL10, and CXCL11 in human monocytes, epithelial cells, and rat alveolar or murine macrophages [34,51,52,53,54,55,56,57,58]. Up-expression of *nos2* and *xdh* induced by the influenza virus infection is mediated by pro-inflammatory cytokines [59,60]. Based on the findings of these studies, we identified the 20 key cytokines/chemokines/ISGs genes for the current study. In our study, we found that the influenza virus FM147 upregulated the expression of *ifnε*, *ifnk*, *ifna2*, *ifnb1*, *ifnγ*, *oas1a*, *oas2*, *oas3*, *mx1*, *il6*, *il1b*, *il12a*, *tnf*, *ccl3*, *ccl4*, *сcl5*, *cxcl9*, *cxcl10*, and *cxcl11* in the mice lungs (Figure 2, Figure 3, Figure 4 and Figure 5). Previous clinical studies have shown that the ORNs-d-М normalized cytokines in patients with genital herpes, hepatitis C, and diabetes [29,30,31]. Therefore, we studied the influence of the ORNs-d-М on up-expression of the cytokines, chemokines, and ISGs-induced influenza virus and found that impairing overexpression of *ifnε*, *ifnk*, *ifna2*, *ifnb1*, *ifnγ*, *oas1a*, *oas2*, *oas3*, *mx1*, *il6*, *il1b*, *il12a*, *tnf*, *ccl3*, *ccl4*, *сcl5*, *cxcl9*, *cxcl10*, and *cxcl11* induced influenza virus infection through the ORNs-d-М that had been injected into mice for prevention and treatment of this infection. These results demonstrated that the ORNs-d-M can normalize cytokines and chemokines levels during influenza virus infection. By inhibiting up-expression of the cytokines, chemokines, and ISGs, the ORNs-d-M can impair overexpression of the *nos2* and *xdh* at influenza virus infection.

The protein complex NF-kB (nuclear factor kappa-light-chain-enhancer of activated B cells) regulates transcription of a large number of genes associated with inflammatory cytokines and downstream ISGs [61,62,63]. Influenza virus infection of airway epithelium cells is dependent on an active NF-kB signaling pathway [64,65]. Cells with low NF-kB activity were virtually resistant to influenza virus infection while activation of the NF-kB signaling pathway by influenza virus infection is not sufficient for allowing infection in these cells [64]. During the influenza virus infection, NF-kB-dependent gene expression is mediated by overexpression of the viral proteins, overproduction ROSs, and activation of the IkB kinase [64]. In the presented study, we found upregulation of the NF-kB1 and the NFKBia-induced influenza virus FM147 (Figure 6a,b). We also estimated an ability of the ORNs-d-M to effect upregulation of the NF-kB1. NFKBia induced the influenza virus and found that the ORNs-d-M, which had been injected into mice for prevention and treatment of the influenza virus infection, have an inhibition effect on the upregulation of the NF-kB1. NFKBia induced the influenza virus. Our results also suggest that, by inhibiting overexpression of the NF-kB1 and NFKBia, the ORNs-d-M can impair influenza-induced overexpression of the cytokines, chemokines, ISGs, and pro-oxidation genes and inhibit influenza virus replication dependent on an active NF-kB signaling pathway [64,65].

The innate immune system is the first stage of protection of an organism against invading pathogens and is associated with a highly conserved host-cell signaling mechanism [66]. Different pattern-recognition receptors are expressed to recognize pathogen-associated molecular patterns, which initiate the signaling cascades inducing cytokine production [66]. The innate immune system recognizes the influenza virus by pattern-recognition receptors such as TLR3 (double-stranded RNA), TLR7 (single-stranded RNA), TLR8 (single-stranded RNA), retinoic acid-inducible gene I (RIG-I) (5′-triphosphate RNA), and the NOD-like receptor family member (various stimuli) [67,68,69]. Activation of TLRs triggers a cascade of signals leading to the activation of NF-kB and the activation of NF-kB dependent production of pro-inflammatory cytokines including IL-6 and TNF-α and the production of type I IFNs [70,71]. In this study, we also found that the influenza virus FM147 induced upregulation of the *tlr3*, *tlr7*, and *tlr8.* Furthermore, in our study, we found that the ORNs-d-M injection for prevention and treatment inhibit the up-expression of *tlr3*, *tlr7*, and *tlr8* induced by the influenza virus infection (Figure 6a,b). These results suggest that the ORNs-d-M can effectively antagonize TLR-3, TLR-7, and TLR-8, inhibit NF-kB activity, and suppress the secretion of the cytokines, chemokines, and pro-oxidants during the influenza virus infection [16,72].

In addition, we evaluated a decreasing replication of the influenza virus by the ORNs-d-M in this in vivo experiment. Both the ORNs-d-M injection for prevention and treatment reduced the infectious titer of influenza virus by 1.4 and 2.2 lgTCID_50_ during the influenza infection (Table 1) [26]. Data analysis of the influenza virus replication and gene expression of innate immune responses shows that a single injections of the ORNs-d-M for the influenza prevention and treatment inhibit partial replication of the influenza virus and normalize the up-expression genes of innate immune responses induced by the influenza infection (for example *infβ1*, *nfkbiα*). In our previous and current studies, we found out that the ORNs-d-M injected for treatment decrease the influenza virus replication better than the ORNs-d-M injected for prevention [26]. Conversely, the ORNs-d-M injection for prevention inhibits the up-expression of genes (*nos2*, *arg2*, *ifnk*, *ifnγ*, *oas3*, *il6*, *il1b*, *il12a*, *ccl3*, *ccl4*, *cxcl9*, *cxcl11*, *nfkbiα*, *tlr3*, *tlr7*, and *tlr8)* induced by the influenza virus better than the ORNs-d-M injection for treatment. The obtained results suggest that, besides inhibiting activity of influenza viral proteins (NA, HA) [26,27], modulating the innate immune response to influenza virus infection by the ORNs-d-M can be another mechanism responsible for their anti-influenza activity.

Complexes of nurture ORNs with d-mannitol are total yeast RNA with a dominant fraction of 3–8 nucleotides modified with d-mannitol. The nurture ORNs bind with low affinity and non-specificity to some target molecules [73]. We believe that in the complexes, there are sequences that bind to the viral protein and change their conformation and activity as well as sequences that bind to the Toll-like receptors. We suggested that different sequences with different actions provide the complexes with a wide range of biological activities. In future research, ORNs-d-M sequences and their binding regions to the Toll-like receptors should be identified and characterized.

## 4. Materials and Methods

The ORNs-d-М complexes were purchased from Goodwill Associates, Washington, DC, USA. Influenza virus A/Fort Monmouth/1/1947-mouse-adapted (H1N1) (FM147) was obtained from the National Virus Collection of D.I. Ivanovsky Institute of Virology (Moscow, Russia) and Madin–Darby canine kidney (MDCK) cells were obtained from the Russian Cell Culture Collection of Russian Academy of Medical Science (Moscow, Russia). BALB/c mice were obtained from the M.M. Shemyakin–Yu.A. Ovchinnikov Institute of Bioorganic Chemistry of the Russian Academy of Sciences (Moscow, Russia). The influenza virus FM147 passed through 15 passages in BALB/c mice. 100**%** mortality of animals is observed 5 days after infection with the influenza virus FM147.

### 4.1. Mouse In Vivo Experiment

The BALB/c mice (14–16 g), 6 to 8 weeks of age, were distributed into five groups as follows: Control—healthy mice (NaCl injection, 0.9%) (*n* = 6), ORNs-d-М control—ORNs-d-М injection into healthy mice as positive control ORNs-d-М (*n* = 6), influenza control—infection of mice with influenza virus as a negative control (*n* = 6), ORNs-d-М + influenza—ORNs-d-М injection 24 h before influenza virus infection and prevention with ORNs-d-М (*n* = 6), and influenza + ORNs-d-М—ORNs-d-М injection 24 h after influenza virus infection and treatment with ORNs-d-М (*n* = 6). To infect each mouse, 100 µL of influenza virus FM147, 4.0 lg LD_50_ diluted in sterile NaCl, 0.9% was administered. For prevention and treatment, each virus-infected mouse was intraperitoneal injected with 100 µL of the ORNs-d-М at concentrations of 15 mg/kg (the minimal active concentration of ORNs-d-М) and diluted in sterile NaCl, 0.9% [26]. After 48 h influenza virus infection (peak of the viral replication), the animals were sacrificed. The research was carried out in the Laboratory of Experimental Chemotherapy for Viral Infections, Gromashevsky L. V. Institute of Epidemiology and Infectious Diseases, NAMSU, Kyiv, Ukraine. The laboratory was certified by the SE “Ukrmetrteststandart” for conducting research “antiviral and virucidal activity of chemistry and plant origin drugs in animals and cell cultures” (№PT 426/14 from 8 December 2014). All procedures that were performed in studies were in accordance with ethical standards (Federalwide Assurance № 00019663).

### 4.2. TCID_50_ Assay

The lungs (100 mg) were homogenized by liquid nitrogen and were soluted into 0.5 mL in sterile NaCl, 0.9%. Then samples were centrifuged at 4000 *g* for 20 min at 4 °C and supernatants were removed. The influenza virus infectious titers were determined in the supernatants using the TCID_50_ assay by the method of Reed–Muench [74]. The infectious titer of the influenza virus was evaluated by the infection of MDCK cells [27].

### 4.3. Real-Time qPCR Assay

Total RNAs were extracted from the lungs (20 mg) by using a NucleoMag 96 RNA Kit (MACHEREY-NAGEL, Duren, Germany) and BeadRetriever system (Invitrogen, Carlsbad, CA, USA), according to the protocol suggested by the manufacturer. RNA integrity was proven by the Microchip electrophoresis system (MCE-202/MultiNA SHIMADZU, Germany), total RNAs were quantified spectrometrically, and RNA purity was assessed by the 260/280 nm ratio on a MaestroNano Pro Micro-Volume MN-913 spectrophotometer (MAESTROGEN, Hsinchu, Taiwan). cDNA was synthesized from every total RNA sample by using a RevertAid H Minus First Standart cDNA Synthesis Kit (Thermo Scientific, Waltham, MA, USA). Random hexamer and oligo (dT)^18^ primers (1:3) were used and the protocol included incubating for 120 min at 42 °C. The reaction was heated to 70 °C for 5 min and chilled on ice. Reverse transcription was conducted using 2 µg total RNA per sample. Messenger mRNA levels of *xdh*, *nos2*, *arg2*, *oas1a*, *oas2*, *oas3*, *mx1*, *rnasel*, *ifnε*, *ifnk*, *ifna2*, *ifnb1*, *ifnγ*, *ccl3*, *ccl4*, *сcl5*, *cxcl9*, *cxcl10*, *cxcl11*, *il6*, *il1b*, *il12a*, *tnf*, *nfkb1*, *nfkbiα*, *tlr3*, *tlr7*, and *tlr8* were quantified by a Thermal Cycler CFX96 Real-Time system (BIO-RAD, Singapore) using 10 µL maxima SYBR Green/Flurescein qPCR masrer mix (Thermo Scientific, Waltham, MA, USA), 0.3 µL cDNA sample, 1.5 µL gene specific primers, and 8.2 µL nuclease-free water. Mice cDNA was amplified with primers listed in Table 2. Quantitative amplification conditions were as follows: denaturation at 95 °C for 10 min, followed by 40 cycles of denaturation at 95 °C for 40 s, annealing at 60 °C for 30 s, and elongation at 72 °C for 30 s. The primers sequenced were designed on GenBank database and were synthesized (Invitrogen, Carlsbad, CA, USA). Average fold change values were determined by the 2^(−ΔΔCt)^ method [75]. The mRNA level of all investigated genes were normalized to *gapdh* mRNA as a control and expression of *gapdh* mRNA were expressed as 100 expression units. The data were presented as mean ± SD.

### 4.4. Lipid Peroxidation Assay

Mouse lungs were homogenized by liquid nitrogen and were soluted into 3 mL of 50 mМ phosphate buffered saline (PBS) (Sigma Aldrich, St. Louis, MO, USA), рН = 7.4. After 100 µL of the homogenized sample was added into 2.5 mL of 0.025 M Tris-HCl, pH = 7.4 (with 0.175 М КCl), 1 mL of 17% of trichloroacetic acid solution was centrifuged at 4000 *g* for 10 min at 4 °C. The protein was measured by the method from Lowry et al. [76]. Endogenous LPO products reacting with 2-thiobarbituric acid (TBA-reactive substances, TBARS) were measured using the SPECORD 210 Plus (Analytik Jena AG, Jena, Germany) at a 532 nm wavelength, which was described by Asakawa & Matsushita [77]. The data were presented as mean ± SD.

### 4.5. Western Blot Analysis

The lungs (20 mg) were lysed in PBS (Sigma Aldrich, St. Louis, MO, USA) with a protease inhibitor cocktail (Sigma Aldrich, Louis, MO, USA). The concentrations of total protein in the lysates were determined by the Bradford protein assay using a Bradford Reagent (Sigma Aldrich, St. Louis, MO, USA), according to the 96 well plate assay protocol suggested by the manufacturer. A total of 30 μg of protein samples was resolved on 10% SDS-PAGE. After the electrophoretic transfer of proteins onto the nitrocellulose membrane (Amersham BioSciences, Buckinghamshire, UK), the membranes were incubated overnight at 4 °C with primary antibodies using the following dilutions: NF-κB 1 (1:500; Rabbit monoclonal; cat. no. 13586; Cell Signaling Technology, Leiden, Netherlands), IκBα (nuclear factor of kappa light polypeptide gene enhancer in B-cells inhibitor, alpha) (1:500; mouse monoclonal; cat. no. sc-1643; Santa Cruz Biotechnology, Dallas, Texas, USA), β-actin (1:20,000; rabbit polyclonal cat. no. A2103; Sigma Aldrich, St. Louis, MO, USA). Afterward, Incubation was performed with horseradish peroxidase (HRP)-conjugated by secondary anti-mouse (1:3000; cat. no. 7076; Cell Signaling Technology, Leiden, Netherlands) and anti-rabbit antibodies (1:3000; cat. no. 7074; Cell Signaling Technology, Leiden, Netherlands) at room temperature for 1 h. The blots were developed and detected with an enhanced chemiluminescence detection kit (West Pico PLUS Chemiluminescent Substrate; Thermo Scientific, Waltham, MA, USA) by following the manufacturer’s instructions. Chemiluminescent signals were captured digitally using a ChemiDoc XRS + system (BIO-RAD, Hercules, CA, USA). Relative levels of the protein were quantified by a densitometric analysis [78]. The volume (intensity) of each band was quantified using Image Lab Software (BIO-RAD, USA). The volume (intensity) of NF-kB1 and NFKBia (IκBα) proteins was normalized to a volume (intensity) of the β-actin protein used as a control.

## 5. Conclusions

The results presented show that, by inhibiting overexpression of the *tlr3*, *tlr7*, and *tlr8* induced by the influenza virus, the ORNs-d-M can impair the upregulation of NF-kB, cytokines, chemokines, ISGs, and pro-oxidation genes induced by the influenza virus. Impairing up-expression of the innate immune genes can be a mechanism of the ORNs-d-M known as an anti-influenza virus drug with effective anti-inflammation activity.

## Figures and Tables

**Table 1:** Decrease of the influenza virus A/Fort Monmouth/1/1947-mouse adapted (H1N1) infectious titer in lungs and weight loss of infected mice after prevention and treatment with the ORNs-d-М.

Group	Infectious Titer of Influenza, lgTCID_50_	Weight loss, g
control	0.0 ± 0.0	13.0 ± 1.3
ORNs-d-М control	0.0 ± 0.0	13.2 ± 0.8
influenza control	6.8 ± 0.12	11.4 ± 1.4
ORNs-d-М + influenza	5.4 ± 0.35	12.0 ± 1.7
Influenza + ORNs-d-М	4.6 ± 0.62	12.5 ± 2.0

**Table 2:** Primers used in this study.

Gene	Primers	Sequence (5′→3′)
*nitric oxide synthase 2, inducible (nos2)*	Forward Reverse	5′-TTT GTG CGA AGT GTC AGT GG-3′ 5′-TCC TTT GAG CCC TTT GTG C-3′
*arginase type II (arg2)*	Forward Reverse	5′-TGA TTG GCA AAA GGC AGA GG-3′ 5′-CTG ACA GCA ACC CTG TAT TAT GTA-3′
*xanthine dehydrogenase (xdh)*	Forward Reverse	5′-CCA AGA TGG TTC AGG TGG C-3′ 5′-TCT GAC AGG CTT CAT AAA TGG C-3′
*2′-5′ oligoadenylate synthetase 1A (oas1a)*	Forward Reverse	5′-ACA GCT CAG AAA AGC CAG G-3′ 5′-CAG TTC TCT TCT ACC TGC TCA AA-3′
*2′-5′ oligoadenylate synthetase 2 (oas2)*	Forward Reverse	5′-CTA TGA TGC ACT AGG TCA ACT GC-3′ 5′-TTC CTT TCA TAC TGT TTG TAC CAG T-3′
*2′-5′ oligoadenylate synthetase 3 (oas3)*	Forward Reverse	5′-CAA AGC GTG GAC TTT GAC G-3′ 5′-ATG GTC TTG TTA CAC TGT TGG TA-3′
*MX dynamin-like GTPase 1 (mx1)*	Forward Reverse	5′-TGC TGT ACT GCT AAG TCC AAA-3′ 5′-GCA GTA GAC AAT CTG TTC CAT CTG-3′
*2′,5′-oligoisoadenylate synthetase-dependent ribonuclease L (rnasel)*	Forward Reverse	5′-GGA CTT GGG AGA ACC GCT AT-3′ 5′-CAT TTT TGT CGA TCT TAG ATG TCC A-3′
*interferon epsilon (ifnε)*	Forward Reverse	5′-CTG GAA TAC GTG GAG TCA CTG-3′ 5′-GAA CCT GAA CAC AAA GAA CAT ACA-3′
*interferon kappa (ifnk)*	Forward Reverse	5′-GGA GTT GGG CAA GTA TTT CTT CA-3′ 5′-CTT GAA GGT GGG TGA TTC TGA TA-3′
*nterferon alpha 2 (ifna2)*	Forward Reverse	5′-CTT ACT CAG CAG ACC TTG AAC C-3′ 5′-CTG CTG CAT CAG ACA GGT TT-3′
*interferon beta 1 (ifnb1)*	Forward Reverse	5′-GAT GCT CCA GAA TGT CTT TCT TGT-3′ 5′-CGA ATG ATG AGA AAG TTC CTG AAG A-3′
*interferon gamma (ifnγ)*	Forward Reverse	5′-AAC TGG CAA AAG GAT GGT GA-3′ 5′-GTT GTT GAC CTC AAA CTT GGC-3′
*chemokine (C-C motif) ligand 3 (ccl3)*	Forward Reverse	5′-GCC ATA TGG AGC TGA CAC C-3′ 5′-TTC TCT TAG TCA GGA AAA TGA CAC C-3′
*chemokine (C-C motif) ligand 4 (ccl4)*	Forward Reverse	5′-AGG GTT CTC AGC ACC AAT G-3′ 5′-TCT TTT GGT CAG GAA TAC CAC AG-3′
*chemokine (C-C motif) ligand 5 (сcl5)*	Forward Reverse	5′-CTC ACC ATC ATC CTC ACT GC-3′ 5′-TGA CAA ACA CGA CTG CAA GA-3′
*chemokine (C-X-C motif) ligand 9 (cxcl9)*	Forward Reverse	5′-CAA AAC TGA AAT CAT TGC TAC ACT GAA-3′ 5′-GGC TGA TCT TCT TTT CCC ATT C-3′
*chemokine (C-X-C motif) ligand 10 (cxcl10)*	Forward Reverse	5′-TGT TGA GAT CAT TGC CAC GAT-3′ 5′-CCT TTT AGA CCT TTT TTG GCT AAA CG-3′
*chemokine (C-X-C motif) ligand 11 (cxcl11)*	Forward Reverse	5′-CTG CTC AAG GCT TCC TTA TGT T-3′ 5′-TTT TTC TAT TGC CTG CAT TAT GAG G-3′
*interleukin 6 (il6)*	Forward Reverse	5′-CTA CCA AAC TGG ATA TAA TCA GGA AAT-3′ 5′-TCT TTT ACC TCT TGG TTG AAG ATA TGA-3′
*interleukin 1 beta (il1b)*	Forward Reverse	5′-TTC ATC TTT GAA GAA GAG CCC AT-3′ 5′-TGG AGA ATA TCA CTT GTT GGT TGA-3′
*interleukin 12a (il12a)*	Forward Reverse	5′-GTG AAG ACG GCC AGA GAA AA-3′ 5′-ACA GGG TCA TCA TCA AAG ACG-3′
*tumor necrosis factor (tnf)*	Forward Reverse	5′-AAA GGG ATG AGA AGT TCC CAA AT-3′ 5′-ACT TGG TGG TTT GCT ACG AC-3′
*nuclear factor kappa B (nfkb1)*	Forward Reverse	5′-GGA CAT GGG ATT TCA GGA TAA CC-3′ 5′-AGA GGT GTC TGA TAC AGG TCA T-3′
*NFKB inhibitor alpha (nfkbiα)*	Forward Reverse	5′-GAG ACT CGT TCC TGC ACT TG-3′ 5′-AAG TGG AGT GGA GTC TGC TG-3′
*toll-like receptor 3 (tlr3)*	Forward Reverse	5′-CCT CTT GAA CAA CGC CCA AC-3′ 5′-AGA GAA AGT GCT CTC GCT GG-3′
*toll-like receptor 7 (tlr7)*	Forward Reverse	5′-ATC CTC TGA CCG CCA CAA TC-3′ 5′-TCA CAT GGG CCT CTG GGA TA-3′
*toll-like receptor 8 (tlr8)*	Forward Reverse	5′-GCC CCC TCA GTC ATG GAT TC-3′ 5′-GAG GGA AGT GCT ATA GTT TGG GG-3′
*glyceraldehyde-3-phosphate dehydrogenase (gapdh)*	Forward Reverse	5′-TGT CGT GGA GTC TAC TGG TGT CTT C-3′ 5′-CGT GGT TCA CAC CCA TCA CAA-3′

## References

[1] Garten R.J., Davis C.T., Russell C.A., Shu B., Lindstrom S., Balish A. (2009). Antigenic and genetic characteristics of swine-origin 2009 A(H1N1) influenza viruses circulating in humans. Science.

[2] Salzberg S. (2008). The contents of the syringe. Nature.

[3] Davlin S.L. (2016). Influenza activity—United States, 2015–2016 season and composition of the 2016–2017 influenza vaccine. MMWR Morb. Mortal. Wkly. Rep..

[4] Hensley S.E. (2014). Challenges of selecting seasonal influenza vaccine strains for humans with diverse pre-exposure histories. Curr. Opin. Virol..

[5] Houser K., Subbarao K. (2015). Influenza vaccines: Challenges and solutions. Cell Host Microbe.

[6] De Clercq E. (2006). Antiviral agents active against influenza a viruses. Nat. Rev. Drug Discov..

[7] Beigel J., Bray M. (2008). Current and future antiviral therapy of severe seasonal and avian influenza. Antivir. Res..

[8] Bright R.A., Shay D.K., Shu B., Cox N.J., Klimov A.I. (2006). Adamantane resistance among influenza A viruses isolated early during the 2005–2006 influenza season in the United States. JAMA.

[9] Moscona A. (2009). Global transmission of oseltamivir-resistant influenza. N. Engl. J. Med..

[10] Sheu T.G. (2011). Dual resistance to adamantanes and oseltamivir among seasonal influenza A (H1N1) viruses: 2008–2010. J. Infect. Dis..

[11] Peasey M., Hall R.J., Sonnberg S. (2010). Pandemic (H1N1) 2009 and Seasonal Influenza A (H1N1) Co-infection, New Zealand, 2009. Emerg. Infect. Dis..

[12] Chertow D.S., Memoli M.J. (2013). Bacterial coinfection in Influenza A. JAMA.

[13] Chen J., Duan M., Zhao Y., Ling F., Xiao K., Li Q., Li B., Lu C., Qi W., Zeng Z. (2015). Saikosaponin a inhibits influenza a virus replication and lung immunopathology. Oncotarget.

[14] Herold S., Steinmueller M., vonWulffen W., Cakarova L., Pinto R., Pleschka S., Mack M., Kuziel W.A., Corazza N., Brunner T. (2008). Lung epithelial apoptosis in influenza virus pneumonia: The role of macrophage-expressed tnf-related apoptosis-inducing ligand. J. Exp. Med..

[15] Ramos I., Fernandez-Sesma A. (2015). Modulating the innate immune response to influenza a virus: Potential therapeutic use of anti-inflammatory drugs. Front. Immunol..

[16] Chang Y.C., Kao W.C., Wang W.Y., Wang W.Y., Yang R.B., Peck K. (2009). Identification and characterization of oligonucleotides that inhibit Toll-like receptor 2-associated immune responses. FASEB.

[17] Wang L., Liu X., Zhang Q., Zhang C., Liu Y., Tu K., Tu J. (2012). Selection of DNA aptamers that bind to four organophosphorus pesticides. Biotechnol. Lett..

[18] Wang R., Zhao J., Jiang T., Kwon Y.M., Lu H., Jiao P., Liao M., Li Y. (2013). Selection and characterization of DNA aptamers for use in detection of avian influenza virus H5N1. J. Virol. Methods.

[19] Zhou J., Swiderski P., Li H., Zhang J., Neff C.P., Akkina R., Rossi J.J. (2009). Selection, characterization and application of new RNA HIV gp 120 aptamers for facile delivery of Dicer substrate siRNAs into HIV infected cells. Nucleic Acids Res..

[20] Feng H., Beck J., Nassal M., Hu K.H. (2011). A SELEXscreened aptamer of human hepatitis B virus RNA encapsidation signal suppresses viral replication. PLoS ONE.

[21] Levchenko S.M., Rebriev A.V., Tkachuk V.V., Dubey L.V., Dubey I.Y., Tkachuk Z.Y. (2013). Studies on the interaction of oligoadenylates with proteins by MALDI-TOF mass spectrometry. Biopolym. Cell.

[22] Skorobogatov O.Y., Lozhko D.N., Zhukov I.Y., Kozlov O., Tkachuk Z.Y. (2014). Study of dephosphorylated 2′-5′-linked oligoadenylates impact on apo-S100A1 protein conformation by heteronuclear NMR and circular dichroism. Biopolym. Cell.

[23] Skorobogatov O.Y., Kukharenko A.P., Kozlov O.V., Dubey I.Y., Tkachuk Z.Y. (2017). 2′-5′-Linked Triadenylates Act as Protein Kinase Activity Modulators. J. Proteom. Bioinform..

[24] Tkachuk Z. (2013). Multiantivirus Compound, Composition and Method for Treatment of Virus Diseases. U.S. Patent.

[25] Melnichuk N., Zarubaev V., Iosyk I., Andreychyn M., Semernikova L., Tkachuk Z. (2018). Pre-Clinical and Clinical Efficiency of Complexes of Oligoribonucleotides with D-Mannitol against Respiratory Viruses. Pharmaceutics.

[26] Tkachuk Z.Y., Rybalko S.L., Zharkova L.D., Starostyla D.B. (2010). Antiinfluenzal activity of drug Nuclex. Rep. Natl. Acad. Sci. Ukr..

[27] Melnichuk N.S., Semernikova L.I., Tkachuk Z.Y. (2017). Complexes of Oligoribonucleotides with d-mannitol inhibit hemagglutinin–glycan interaction and suppress influenza A virus H1N1 (A/FM/1/47) infectivity In Vitro. Pharmaceuticals.

[28] Tkachuk Z.Y., Tkachuk V.V., Tkachuk L.V. (2006). The study on membrane-stabilizing and anti-inflammatory actions of yeast RNA in vivo and in vitro. Biopolym. Cell.

[29] Tkachuk Z., Chercasova V., Frolov V. (2012). Dynamics of indexes of interferon status of blood of patients with genital herpes at application of nuclex. Ukr. Morphol. Almanac..

[30] Tkachuk Z.Y., Frolov V.M., Sotska Y.A., Kruglova O.V. (2012). Nuclex therapy for patients with chronic hepatitis C. Int. J. Immunol. Stud..

[31] Zelyoniy I.I., Tkachuk Z.Y., Afonin D.N., Tiutiunnyk A.A. (2013). Influence of Preparation Nucleх on the Cytokine Profile of the Patients with Diabetes Type 2 and Neuropathic Form of Diabetic Foot. J. Diabetes Res..

[32] Akaike T. (2001). Role of free radicals in viral pathogenesis and mutation. Rev. Med. Virol..

[33] Perrone L.A., Belser J.A., Wadford D.A., Katz J.M., Tumpey T.M. (2013). Inducible Nitric Oxide Contributes to Viral Pathogenesis Following Highly Pathogenic Influenza Virus Infection in Mice. Infect. Diseases.

[34] Zou W., Chen D., Xiong M., Zhu J., Lin X., Wang L., Zhang J., Chen L., Zhang H., Chen H. (2013). Insights into the increasing virulence of the swine-origin pandemic H1N1/2009 influenza virus. Sci. Rep..

[35] Dawson T.C., Beck M.A., Kuziel W.A., Henderson F., Maeda M. (2000). Contrasting effects of CCR5 and CCR2 deficiency in the pulmonary inflammatory response to influenza A virus. Am. J. Pathol..

[36] Akaike T., Ando M., Oda T., Doi T., Ijiri S., Araki S., Maeda H. (1990). Dependence on O_2_ Generation by Xanthine Oxidase of Pathogenesis of Influenza Virus Infection in Mice. J. Clin. Investig..

[37] Lucas R., Czikora I., Sridhar S., Zemskov E.A., Oseghale A., Circo S. (2013). Arginase 1: An unexpected mediator of pulmonary capillary barrier dysfunction in models of acute lung injury. Front. Immunol..

[38] Nikam S.V., Nikam P.S., Chandrashekar M.R., Kalsad S.T., Jnaneshwara K.B. (2010). Role of lipid peroxidation, glutathione and antioxidant enzymes in H1N1 Influenza. Biomed. Res..

[39] Tkachuk Z. (2001). Method of Protecting Erythricytes, in Particular for Improvement of Blood Cytopenia. U.S. Patent.

[40] Diaz M.O., Bohlander S., Allen G. (1996). Nomenclature of the human interferon genes. J. Interf. Cytokine Res..

[41] Young H.A. (1996). Regulation of interferon-γ gene expression. J. Interf. Cytokine Res..

[42] Stark G.R., Kerr I.M., Williams B.R., Silverman R.H., Schreiber R.D. (1998). How cells respond to interferons. Annu. Rev. Biochem..

[43] Pestka S., Langer J.A., Zoon K.C., Samuel C.E. (1987). Interferons and their actions. Annu. Rev. Biochem..

[44] Samuel C.E. (1988). Mechanisms of the Antiviral Actions of IFN. Prog. Nucleic Acid Res. Mol. Biol..

[45] Pestka S., Krause C.D., Walter M.R. (2004). Interferons, interferon-like cytokines, and their receptors. Immunol. Rev..

[46] Xing Z., Harper R., Anunciacion J., Yang Z., Gao W., Qu B., Guan Y., Cardona C.J. (2011). Host immune and apoptotic responses to avian influenza virus H9N2 in human tracheobronchial epithelial cells. Am. J. Respir. Cell Mol. Biol..

[47] Chan M.C., Cheung C.Y., Chui W.H., Tsao S.W., Nicholls J.M., Chan Y.O., Chan R.W., Long H.T., Poon L.L., Guan Y. (2005). Proinflammatory cytokine responses induced by influenza A (H5N1) viruses in primary human alveolar and bronchial epithelial cells. Respir. Res..

[48] Haller O., Staeheli P., Kochs G. (2009). Protective role of interferon-induced Mx GTPases against influenza viruses. Rev. Sci. Technol..

[49] Samuel C.E. (2001). Antiviral actions of interferons. Clin. Microbiol. Rev..

[50] Trinchieri G. (2010). Type I interferon: Friend or foe?. J. Exp. Med..

[51] Matsukura S., Kokubu F., Noda H., Tokunaga H., Adachi M. (1996). Expression of IL6, IL-8, and RANTES on human bronchial epithelial cells, NCI-H292, induced by influenza virus A. J. Allergy Clin. Immunol..

[52] Adachi M., Matsukura S., Tokunaga H., Kokubu F. (1997). Expression of cytokines on human bronchial epithelial cells induced by influenza virus A. Int. Arch. Allergy Immunol..

[53] Nain M., Hinder F., Gong J.H., Schmidt A., Bender A., Sprenger H., Gemsa D. (1990). Tumor necrosis factor-alpha production of influenza A virus-infected macrophages and potentiating effect of lipopolysaccharides. J. Immunol..

[54] Julkunen I., Melen K., Nyqvist M., Pirhonen J., Sareneva T., Matikainen S. (2000). Inflammatory responses in influenza a virus infection. Vaccine.

[55] Julkunen I., Sareneva T., Pirhonen J., Ronni T., Melen K., Matikainen S. (2001). Molecular pathogenesis of influenza A virus infection and virus-induced regulation of cytokine gene expression. Cytokine Growth Factor Rev..

[56] Lam W.Y., Yeung A.C., Chu I.M., Chan P.K. (2010). Profiles of cytokine and chemokine gene expression in human pulmonary epithelial cells induced by human and avian influenza viruses. Virol. J..

[57] Lam W.Y., Yeung A.C., Chan P.K. (2011). Apoptosis, cytokine and chemokine induction by non-structural 1 (NS1) proteins encoded by different influenza subtypes. Virol. J..

[58] Bao Y., Gao Y., Shi Y., Cui X. (2017). Dynamic gene expression analysis in a H1N1 influenza virus mouse pneumonia model. Virus Genes.

[59] Akaike T., Noguchi Y., Ijiri S., Setoguchi K., Suga M., Zheng Y.M., Dietzschold B., Maeda H. (1996). Pathogenesis of influenza virusinduced pneumonia: Involvement of both nitric oxide and oxygen radicals. Proc. Natl. Acad. Sci. USA.

[60] Akaike T., Maeda H. (2000). Nitric oxide and virus infection. Immunology.

[61] Kopp E.B., Ghosh S. (1995). NF-kappa B and rel proteins in innate immunity. Adv. Immunol..

[62] Baeuerle P.A., Baltimore D. (1996). NF-kappa B: Ten years after. Cell.

[63] Baeuerle P.A., Henkel T. (1994). Function and activation of NF-kappa B in the immune system. Annu. Rev. Immunol..

[64] Flory E., Kunz M., Scheller C., Jassoy C., Stauber R., Rapp U.R., Ludwig S. (2000). Influenza virus-induced NF-kB-dependent gene expression is mediated by overexpression of viral proteins and involves oxidative radicals and activation of IkB kinase. J. Biol. Chem..

[65] Nimmerjahn F., Dudziak D., Dirmeier U., Hobom G., Riedel A., Schlee M., Staudt L.M., Rosenwald A., Behrends U., Bornkamm G.W. (2004). Active NF-kappaB signalling is a prerequisite for influenza virus infection. J. Gen. Virol..

[66] Akira S., Uematsu S., Takeuchi O. (2006). Pathogen recognition and innate immunity. Cell.

[67] Le Goffic R., Balloy V., Lagranderie M., Alexopoulou L., Escriou N., Flavell R., Chignard M., Si-Tahar M. (2006). Detrimental contribution of the Toll-like receptor (TLR)3 to influenza A virus-induced acute pneumonia. PLoS Pathog..

[68] Diebold S.S., Kaisho T., Hemmi H., Akira S., Reis e Sousa C. (2004). Innate antiviral responses by means of TLR7-mediated recognition of single-stranded RNA. Science.

[69] Pang I.K., Iwasaki A. (2012). Control of antiviral immunity by pattern recognition and the microbiome. Immunol. Rev..

[70] Kawai T., Akira S. (2011). Toll-like Receptors and Their Crosstalk with Other Innate Receptors in Infection and Immunity. Immunity.

[71] Alexopoulou L., Holt A.C., Medzhitov R., Flavell R.A. (2001). Recognition of double-stranded RNA and activation of NF-kappaB by Toll-like receptor 3. Nature.

[72] Li Z., Li L., Zhou H., Zeng L., Chen T., Chen Q., Zhou B., Wang Y., Chen Q., Hu P. (2017). Radix isatidis Polysaccharides Inhibit Influenza a Virus and Influenza A Virus-Induced Inflammation via Suppression of Host TLR3 Signaling In Vitro. Molecules.

[73] Vivcharyk M., Iakhnenko M., Levchenko S., Chernykh S., Tkachuk Z. Monitoring of Interferon-α (peg) conformational changes caused by yeast RNA. Proceedings of the 7th International Conference Physics of Liquid Matter: Modern Problems (PLM MP).

[74] Reed L.J., Muench H. (1938). A simple method of estimating fifty percent endpoints. Am. J. Hyg..

[75] Livak K.J., Schmittgen T.D. (2001). Analysis of relative gene expression data using real-time quantitative PCR and the 2(-delta delta C(T)) method. Methods.

[76] Lowry O.H., Rosebrough N.J., Farr A.L., Randall R.J. (1951). Protein measurement with the Folin phenol reagent. J. Biol. Chem..

[77] Asakawa T., Matsushita S. (1980). Thiobarbituric acid test for detecting lipid peroxides. Lipids.

[78] Schmidt G., Amiraian K., Frey H., Stevens R.W., Berns D.S. (1987). Densitometric analysis of Western blot (immunoblot) assays for human immunodeficiency virus antibodies and correlation with clinical status. J. Clin. Microbiol..

